# 31. Development a Novel Score (SAD-60) for Predicting Mortality in Hospitalised Patients with COVID-19 Pneumonia: A Multicenter Retrospective Study of 1013 Patients

**DOI:** 10.1093/ofid/ofab466.031

**Published:** 2021-12-04

**Authors:** Serkan Surme, Hatice Kubra Karanalbant, Gulsah Tuncer, Osman Bayramlar, Betul Copur, Esra Zerdali, İnci Yilmaz Nakir, Meltem Yazla, Ahmet Buyukyazgan, Ayse Kurt Cinar, Yesim Kurekci, Mustafa Alkan, Yusuf Emre Ozdemir, Gonul Sengoz, Filiz Pehlivanoglu

**Affiliations:** 1 Haseki Training and Research Hospital, Istanbul, Istanbul, Turkey; 2 Bakirkoy District Health Directorate, Istanbul, Istanbul, Turkey; 3 Bahcelievler State Hospital, Istanbul, Turkey, Istanbul, Istanbul, Turkey; 4 Bayrampasa State Hospital, Istanbul, Turkey, Istanbul, Istanbul, Turkey; 5 Arnavutkoy State Hospital, Istanbul, Turkey, Istanbul, Istanbul, Turkey; 6 Gaziosmanpasa Training and Research Hospital, Istanbul, Istanbul, Turkey; 7 Bakirkoy Sadi Konuk Training and Research Hospital, Istanbul, Istanbul, Turkey

## Abstract

**Background:**

We aimed to explore a novel risk score to predict mortality in hospitalised patients with COVID-19 pneumonia. In additoon, we compared the accuracy of the novel risk score with CURB-65, qSOFA and NEWS2 scores.

**Methods:**

The study was conducted in hospitalised patients with laboratory and radiologically confirmed COVID-19 pneumonia between November 1, 2020 and November 30, 2020. In this retrospective multicenter study. independent predictors were identified using multivariate logistic regression analysis. A receiver operating characteristics (ROC) analysis with area under the curve (AUC) was used to evaluate the performance of the novel score. The optimal cut‐off points of the candidate variables were calculated by the Youden’s index of ROC curve. Mortality was defined as all cause in-hospital death.

**Results:**

A total of 1013 patients with COVID-19 were included. The mean age was 60,5 ±14,4 years, and 581 (57,4%) patients were male. In-hospital death was occured in 124 (12,2%) patients. Multivariate analysis revealed that peripheral capillary oxygen saturation (SpO2), albumin, D-dimer, and age were independent predictors for mortality (Table). A novel scoring model was named as SAD-60 (**S**pO2, **A**lbumin, **D**-dimer, **≥60** years old). SAD-60 score (0,776) had the highest AUC compared to CURB-65 (0,753), NEWS2 (0,686), and qSOFA (0,628) scores (Figure).

**Conclusion:**

We demonstrated that SAD-60 score had a promising predictive capacity for mortality in hospitalised patients with COVID-19.

Univariate and multivariate analysis of factors predicting mortality

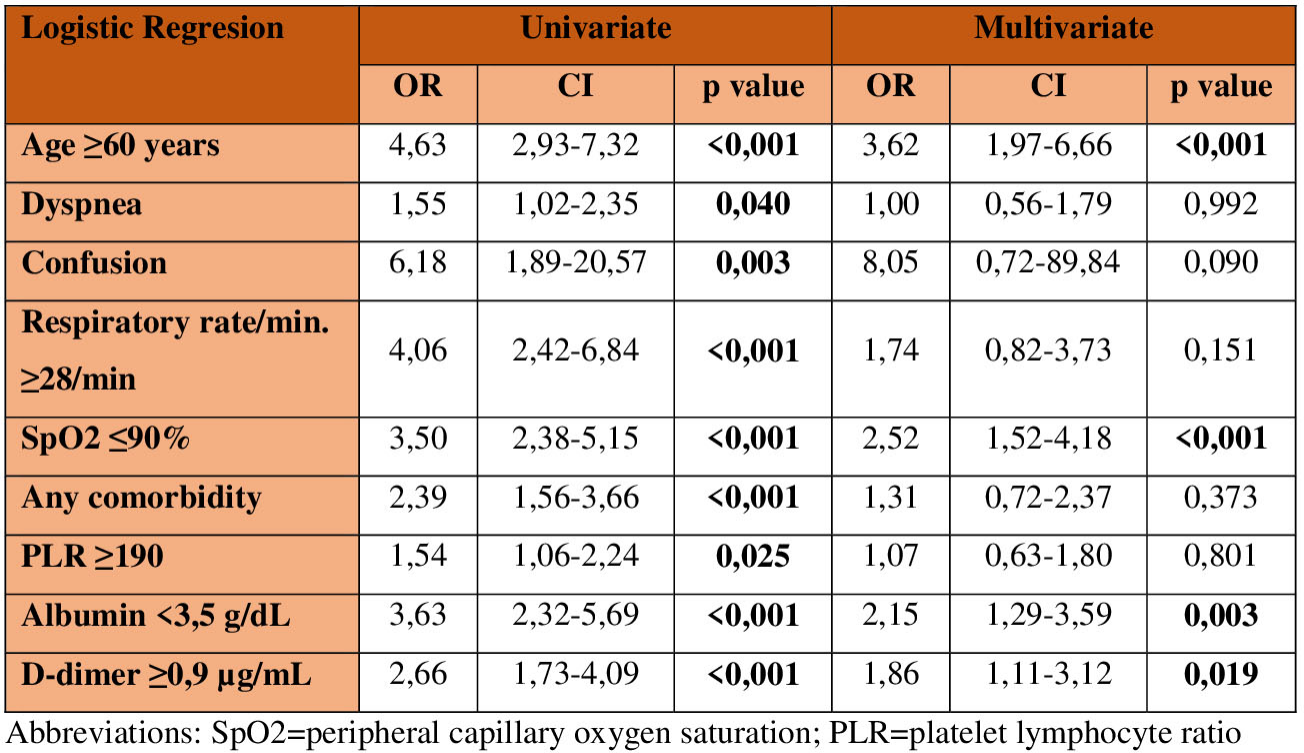

Comparison of CURB-65, qSOFA, NEWS-2 and SAD-60 for predicting pneumonia mortality in hospitalised patients with COVID-19 by ROC analysis

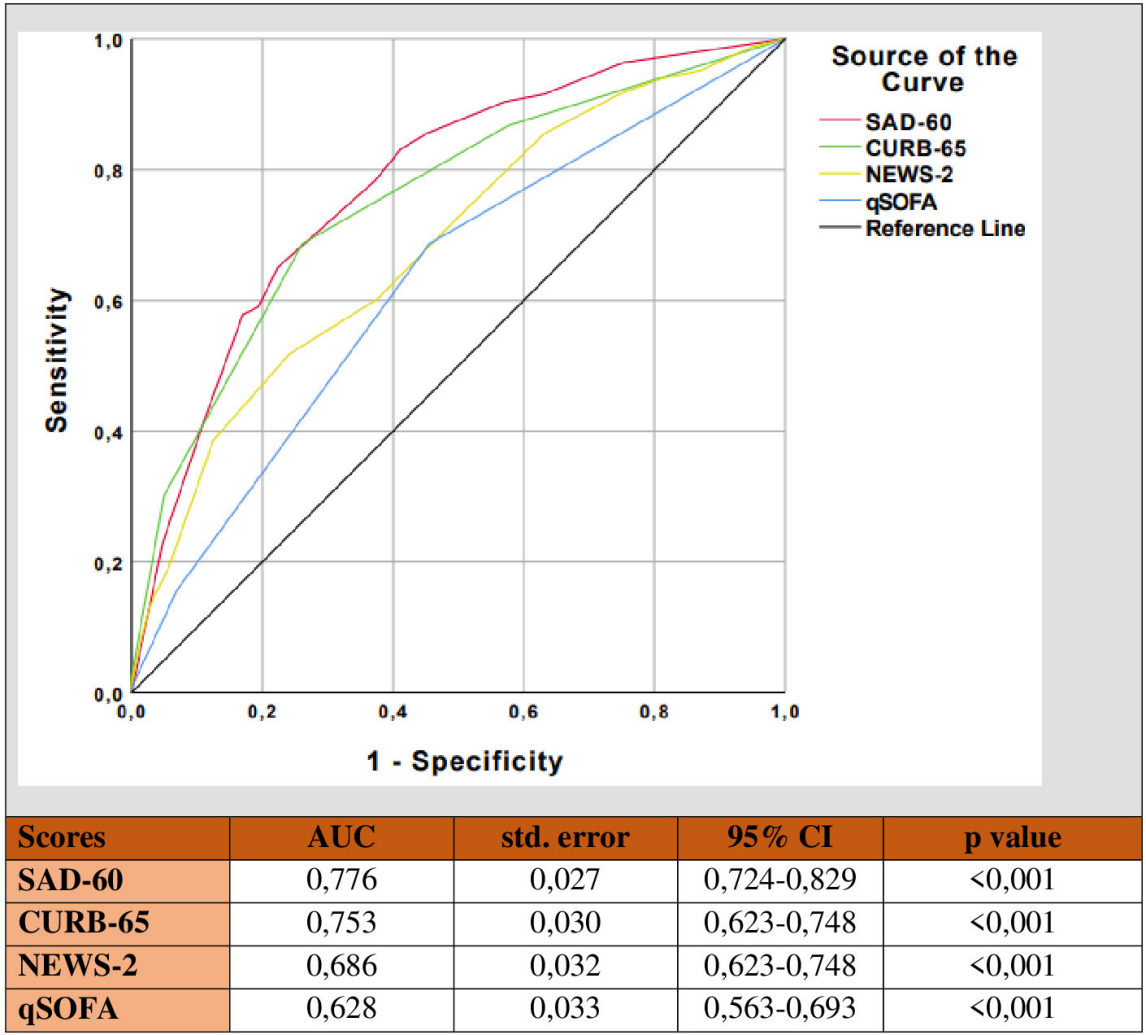

**Disclosures:**

**All Authors**: No reported disclosures

